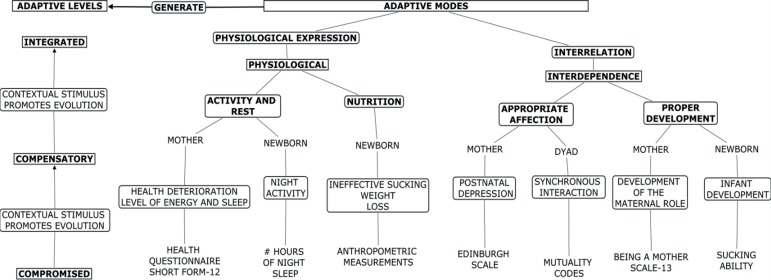# Erratum

**DOI:** 10.1590/1518-8345.0000.3244

**Published:** 2019-12-05

Regarding the article “Assessment of nursing care using indicators generated by software”, with DOI number: 10.1590/0104-1169.0177.2547”, published in Rev. Latino-Am. Enfermagem, 2015;23(2)6:234-41, page 1:

Where was written:

“Assessment of nursing care using indicators generated by software^1^

Ana Paula Souza Lima^2^

Tânia Couto Machado Chianca^3^

Meire Chucre Tannure^4^

^1^ Paper extrated from master’s thesis “Avaliação da assistência de enfermagem através de indicadores gerados por um software”, presented to Universidade Federal de Minas Gerais, Belo Horizonte, MG, Brazil. 

^2^ MSc, Nurse, Hospital da Polícia Militar, Belo Horizonte, MG, Brazil. 

^3^ PhD, Full Professor, Escola de Enfermagem, Universidade Federal de Minas Gerais, Belo Horizonte, MG, Brazil. 

^4^ PhD, Adjunct Professor, Escola de Enfermagem, Pontifícia Universidade Católica de Minas Gerais, Belo Horizonte, MG, Brazil.”

Now read:

“Assessment of nursing care using indicators generated by software*

Ana Paula Souza Lima^1^

Tânia Couto Machado Chianca^2^

Meire Chucre Tannure^3^

* Paper extrated from master’s thesis “Avaliação da assistência de enfermagem através de indicadores gerados por um software”, presented to Universidade Federal de Minas Gerais, Belo Horizonte, MG, Brazil. Supported by Fundação de Amparo à Pesquisa do Estado de Minas Gerais (FAPEMIG), Brazil, grant # APQ-01785-11.

^1^ MSc, Nurse, Hospital da Polícia Militar, Belo Horizonte, MG, Brazil. 

^2^ PhD, Full Professor, Escola de Enfermagem, Universidade Federal de Minas Gerais, Belo Horizonte, MG, Brazil. 

^3^ PhD, Adjunct Professor, Escola de Enfermagem, Pontifícia Universidade Católica de Minas Gerais, Belo Horizonte, MG, Brazil.”

Regarding the article “Impact of urinary incontinence on the quality of life of individuals undergoing radical prostatectomy”, with DOI number: 10.1590/1518-8345.2757.3131, published in Rev. Latino-Am. Enfermagem, 2019;27:e3131, page 1:

Where was written:

“Luciana Regina Ferreira Pereira da Mata^1^

^1^ Universidade Federal de Minas Gerais, Escola de Enfermagem, Belo Horizonte, MG, Brazil.”

**How to cite this article**

Bernardes MFVG, Chagas SC, Izidoro LCR, Veloso DFM, Chianca TCM, Mata LRFP. Impact of urinary incontinence on the quality of life of individuals undergoing radical prostatectomy. Rev. Latino-Am. Enfermagem. 2019;27:e3131. [Access 
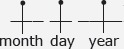
; Available in: 
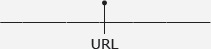
. DOI: http://dx.doi.org/10.1590/1518-8345.2757.3131.

Now read:

“Luciana Regina Ferreira da Mata^1^

^1^ Universidade Federal de Minas Gerais, Escola de Enfermagem, Belo Horizonte, MG, Brazil.”

**How to cite this article**

Bernardes MFVG, Chagas SC, Izidoro LCR, Veloso DFM, Chianca TCM, Mata LRF. Impact of urinary incontinence on the quality of life of individuals undergoing radical prostatectomy. Rev. Latino-Am. Enfermagem. 2019;27:e3131. [Access 
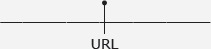
; Available in: 
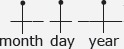
. DOI: http://dx.doi.org/10.1590/1518-8345.2757.3131.

Regarding the article “Effects of interprofessional education on teamwork and on knowledge chronic conditions management”, with DOI number: 10.1590/1518-8345.3095.3203, published in Rev. Latino-Am. Enfermagem, 2019;27:e3203, page 1:

Where was written:

“Effects of interprofessional education on teamwork and on knowledge chronic conditions management*”

Now read:

“Effect of interprofessional education on teamwork and on knowledge of chronic conditions management*”

Regarding the article “Effect of infant stimulation on the adaptation to birth: a randomized trial”, with DOI number: 10.1590/1518-8345.2896.3176, published in Rev. Latino-Am. Enfermagem, 2019;27:e3176, page 3:

Where was written:
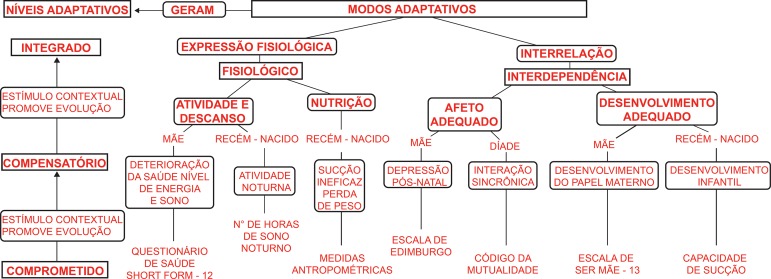


Now read: